# A Rare Case of Granulomatosis With Polyangiitis Presenting as Retroperitoneal Fibrosis in the Peri-Iliac Region Causing Hydronephrosis

**DOI:** 10.7759/cureus.17295

**Published:** 2021-08-18

**Authors:** Kristijan Šoštarič, Tina Lovrec Krstić, Aleš Slanič, Primož Caf

**Affiliations:** 1 Radiology, University Medical Centre Maribor, Maribor, SVN

**Keywords:** retroperitoneal fibrosis, granulomatosis with polyangiitis, wegener's granulomatosis, hydronephrosis, gpa

## Abstract

Wegener’s granulomatosis, now more commonly referred to as granulomatosis with polyangiitis (GPA), is a rare, idiopathic, systemic inflammatory disease, most commonly involving the respiratory tract, kidneys, and sinonasal region. The condition affects small and medium-sized blood vessels, such as arteries, arterioles, venules, and capillaries. Some cases of the disease presenting as retroperitoneal fibrosis and/or affecting the aorta have been reported. Although advances in the treatment of GPA have contributed to a decline in mortality, early diagnosis is still of vital importance due to the possible complications of the disease.

Here, we present the case of a 69-year-old man with acute-onset abdominal pain. Ultrasound of the abdomen showed left-sided hydronephrosis. Computed tomography detected cavitating pulmonary lesions and peri-iliac alterations caused by retroperitoneal fibrosis with involvement of the crossing ureter. Laboratory results revealed high antineutrophil cytoplasmic antibody levels and high inflammatory markers. A lung biopsy performed via bronchoscopy revealed necrotizing granulomas and solidified the diagnosis of GPA both in the lung and the peri-iliac region. Treatment with immunosuppressive agents and glucocorticoids was initiated. A follow-up after two months showed regression of the pulmonary lesions and partial resolution of the hydronephrosis as well as reduced inflammatory markers in the blood tests.

## Introduction

Granulomatosis with polyangiitis (GPA), previously referred to as Wegener’s granulomatosis, is a rare, idiopathic, systemic, auto-inflammatory disease. Typically, as the name suggests, the disease presents with necrotizing granulomatous inflammation coupled with small and medium-sized blood vessel vasculitis caused by antineutrophil cytoplasmic antibodies (ANCA) antibodies [1]. GPA is a rare disease with an estimated prevalence of 2.4-15.6 per 100,000 and a yearly incidence of 0.3-1.4 per 100,000. Both genders are affected equally and at all ages, with the mean age of onset in the fourth decade [2,3]. The etiology of GPA is under intense research but remains partially unknown. Some environmental factors and exposure to microbial infections appear to be relevant in the pathogenesis of the disease. These external agents may be the reason for a nonspecific immune reaction with increased cytokine levels, which further elevate ANCA levels and lead to cell destruction. While advances in the treatment of GPA have contributed to a decline in mortality, early diagnosis remains crucial due to the possible complications of the disease [1].

Early diagnosis remains a challenge because of the different nonspecific clinical manifestations patients present with during the early stages of the disease. These include, but are not limited to, rhinorrhea, oral ulcers, chronic sinusitis, and dyspnea [1]. When GPA is fully developed, at least two of the following criteria are met: inflammation in the nasal and oral region with ulcers or nasal discharge, abnormal chest radiographs with nodules, cavities or infiltrates, urinary sediment showing microhematuria, and histologically confirmed granulomatous inflammation in either vascular walls or perivascular area [4].

The clinical manifestations range from predominantly granulomatous involvement in the respiratory tract to severe multiorgan vasculitis. The most commonly affected organs are the lungs and kidneys, with lung involvement present in over 90% of cases [5,6]. Literature shows rare cases of GPA affecting large vessels and other structures such as the aorta and retroperitoneal tissue. These changes are often overlooked during clinical evaluation because of their rarity, but the associated risks of dissection or obstruction of local structures by the inflammation make an early diagnosis paramount. Advances in the treatment of GPA have contributed to a decline in mortality and complications. Many potential medications have been considered for GPA, but corticosteroids and cyclophosphamide remain the first-line treatment for achieving and sustaining a state of disease remission [6,7].

## Case presentation

We present the case of a 69-year-old patient with no known chronic comorbidities. In 2018, he underwent a transurethral prostate resection because of benign prostatic hyperplasia. No occupational exposure to asbestos was reported. He was brought to the Emergency Room with acute-onset abdominal pain which reportedly worsened during movement and radiated from his left lumbar region to his groin, with a Visual Analog Scale score of about 7/10. An examination showed diffuse tenderness of the abdomen without signs of peritoneal irritation and was otherwise unremarkable. The patient did not report vomiting or notice any blood in his stool, which was confirmed by a negative hematest. However, he mentioned moderate constipation in the past as well as that week. The patient was hemodynamically stable on admission, afebrile, and did not present any other symptoms.

**Figure 1:**
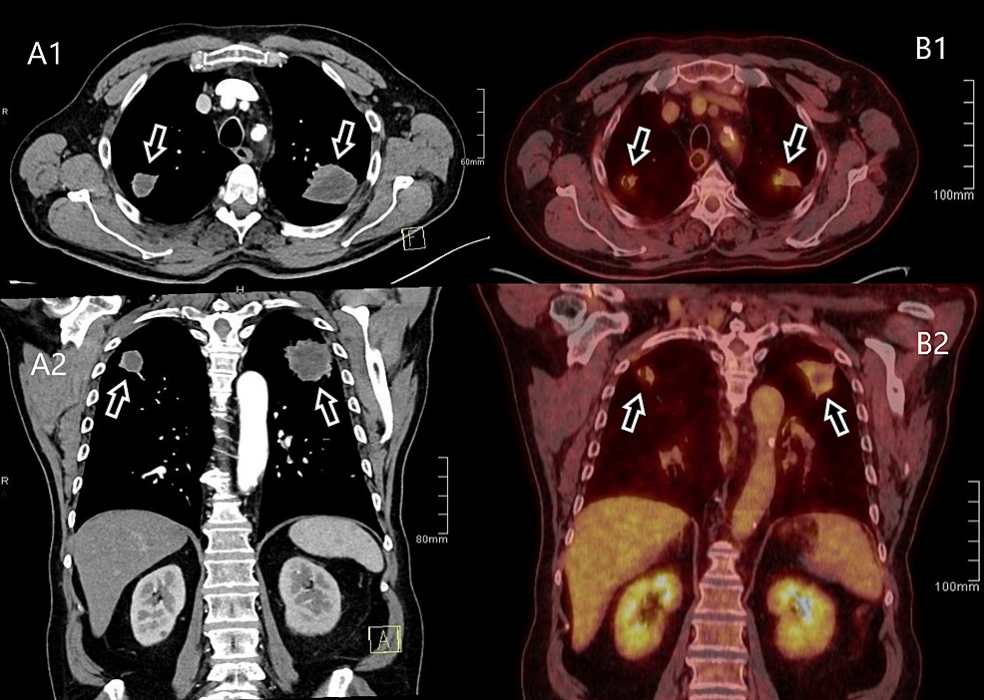
CT of the chest in the axial and coronal planes when the patient was first admitted to the hospital (A1, A2), and a follow-up PET-CT in the axial and coronal planes after two months of treatment, showing a decrease in lesion size (B1, B2). Lesions are marked with arrows. CT: computed tomography; PET-CT: positron emission tomography-computed tomography

Blood tests showed an elevated C-reactive protein (CRP) (50 mg/L) and a deterioration in renal function (creatinine of 158 µmol/L). Point-of-care ultrasound of the abdomen showed unilateral hydronephrosis. Because the patient mentioned constipation, an X-ray was performed to rule out ileus, which showed nothing of significance in the abdomen, but caught an incidental mass at the base of the lungs. Computed tomography (CT) of the chest revealed bilateral cavitating lesions in the lungs (Figure 1). Physicians were unsure whether the lesions were metastases with a primary tumor of the lungs or GPA. Radiological examinations were performed to gain additional information.

**Figure 2:**
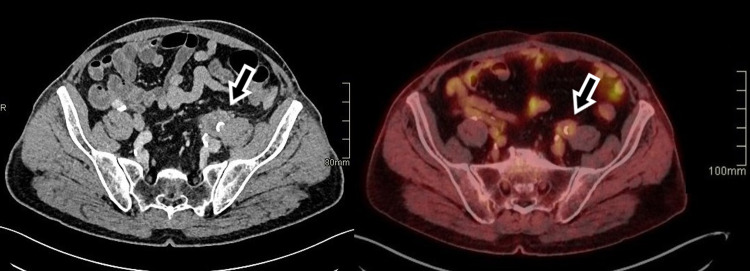
CT of the abdomen in the axial plane when the patient was first admitted to the hospital (A), and a follow-up PET-CT in the axial plane after two months of treatment (B). The retroperitoneal fibrosis is marked with arrows, showing a decrease in mass size. CT: computed tomography; PET-CT: positron emission tomography-computed tomography

**Figure 3:**
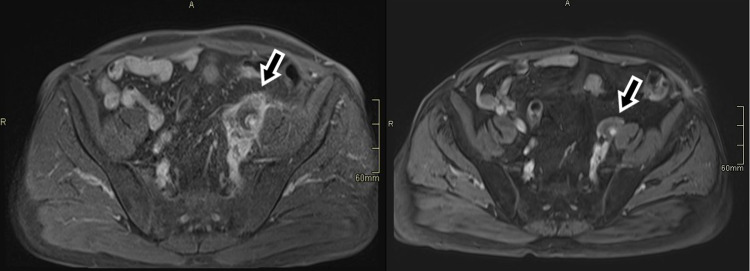
T1 sequence MRI of the abdomen in the axial plane when the patient was first admitted to the hospital (A), and a follow-up after two months of treatment (B). The retroperitoneal fibrosis is marked with arrows. MRI: magnetic resonance imaging

CT of the abdomen revealed dilated two-thirds of the left ureter with hydronephrosis, as well as a pathologic soft tissue mass around the ureter and left external iliac artery (Figure 2). The most likely differential diagnosis at the time was tumor or alterations of inflammatory etiology. Magnetic resonance (MR) angiography of the abdomen and pelvis showed retroperitoneal fibrosis of the left iliac abdominal area with a thickened wall of the external iliac artery and a 50% occlusion of the blood vessel (Figure 3). Immunology tests showed positive antineutrophil cytoplasmic antibodies (c-ANCA) and anti-proteinase 3 (PR3-ANCA) antibodies (362 kRU/L). A lung biopsy performed via bronchoscopy revealed necrotizing granulomas and solidified the diagnosis of GPA involving both the lung as well as the peri-iliac region, with consequent retroperitoneal fibrosis.

**Figure 4:**
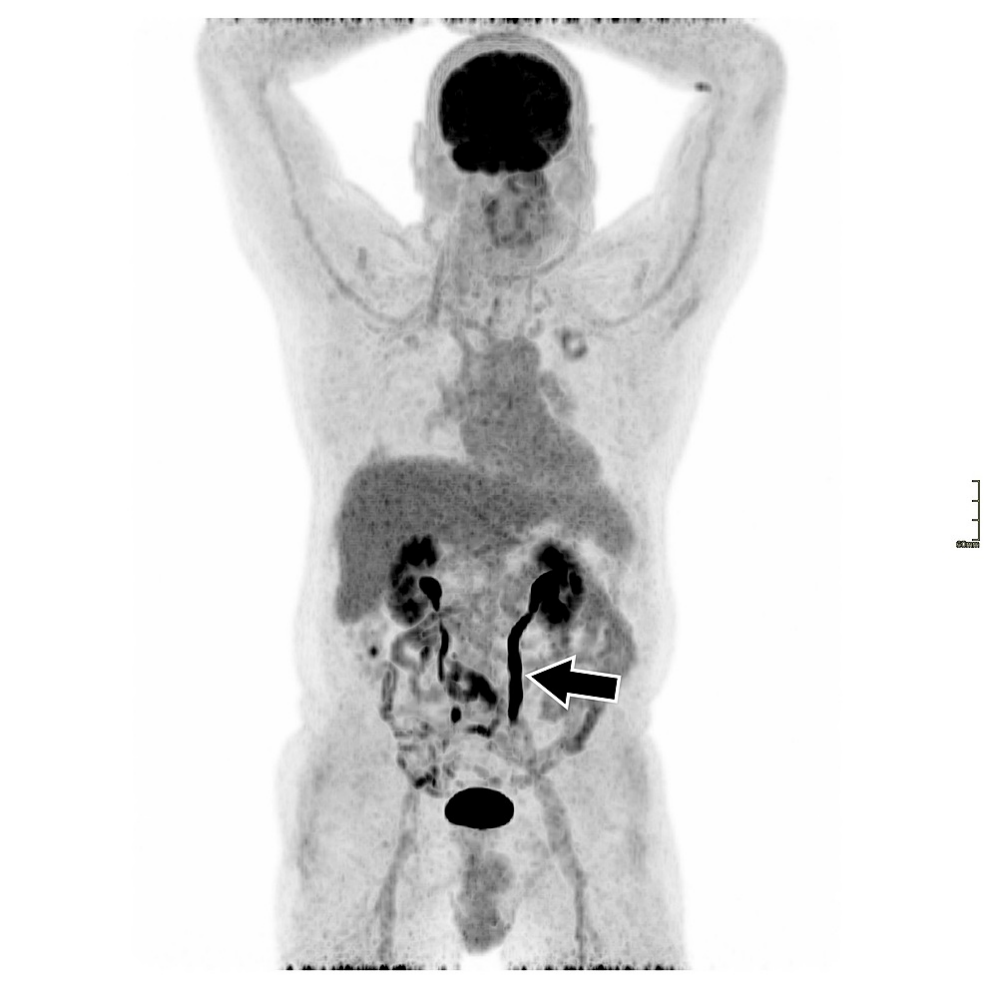
PET-CT whole body MIP after two months of treatment. The arrow is showing a dilated left ureter. PET-CT: positron emission tomography-computed tomography; MIP: maximum intensity projection

The patient was moved to the rheumatology department and started treatment with immunosuppressive agents and glucocorticoids. After two months, the patient has significantly improved both clinically and during examinations. Acute-phase reactants have normalized (CRP <3.0 mg/dL). Follow-up imaging studies confirm a substantial reduction in the size of pulmonary lesions as well as a notable decrease in the size of the retroperitoneal mass. Positron emission tomography-computed tomography shows lower activity levels in the affected areas (Figures 1-3). Maximum intensity projection imaging shows a partial resolution of the hydronephrosis and a fully functioning left kidney. The ureter remains dilated (Figure 4).

## Discussion

We have presented the case of a 69-year-old patient with acute-onset abdominal pain caused by GPA-associated peri-iliac retroperitoneal fibrosis resulting in a compression of local structures and hydronephrosis. Because this presentation is atypical, it can delay the correct diagnosis and prolong the time without adequate treatment. The disease progression is associated with potentially dangerous complications, and achieving a quick remission should be the primary goal. In our case, radiological scans and blood tests guided the physicians in the right direction and reduced the time needed to initiate treatment.

GPA frequently affects the eye/nose/throat region, with involvement in up to 88% of documented cases. The symptoms include sinusitis, nasal obstruction, epistaxis, scleritis, ductal lacrimal obstruction, and otitis media. Complications can develop in the form of nasal perforations and saddle nose defects, which are considered to be specific signs of GPA, but develop later as the disease progresses [8]. Most patients develop pulmonary symptoms, most commonly chronic cough, pulmonary infiltration, pulmonary nodules, chest pain, lung masses, or organized pneumonia. If the disease remains untreated, the symptoms can escalate to tracheal stenosis and pulmonary hemorrhage [9]. Untreated GPA eventually leads to glomerulonephritis seen in up to 80% of documented patients. Other clinical manifestations of GPA include, but are not limited to, cardiovascular symptoms such as thrombotic events and pericarditis, musculoskeletal symptoms such as arthralgia and muscle pain, cutaneous symptoms such as purpura and cutaneous vasculitis, or gastrointestinal symptoms such as abdominal pain, nausea, dyspepsia, vomiting, and diarrhea [10].

Retroperitoneal fibrosis is a rare condition with a complex confirmation of diagnosis. About 70% of known cases are of idiopathic origin. Usually, a prolonged follow-up period is required along with a complex workup to exclude any underlying malignancy. The underlying causes of retroperitoneal fibrosis as well as its connection to other diseases have not yet been broadly described and are still being researched. Based on a Finnish retrospective study performed in 2004, idiopathic retroperitoneal fibrosis has an incidence of 1/10^6^ person-years and a prevalence of 1.4 × 10^5^ inhabitants. The authors concluded that their chosen methodology was suboptimal as the data were obtained from the hospital discharge diagnostic codes and some cases were not properly labeled and consequently missed [11]. A second study published in 2009 by Van Bommel et al. collected all documented retroperitoneal fibrosis cases at the Albert Schweitzer Hospital in the Netherlands. They looked at a period of 10 years and concluded that the annual incidence of retroperitoneal fibrosis was 1.3/10^5^ inhabitants, which is more than 10 times higher compared to the Finnish study. More data are needed to further narrow down the incidence and prevalence of the disease as well as establish better diagnostic criteria [12].

When retroperitoneal fibrosis is confirmed, physicians must exclude drugs, malignancy, infections, radiation therapy, surgery, or asbestos exposure. Rarer causes have been described in the literature, ranging from immunoglobulin G4 (IgG4)-related disease to systemic vasculitis involving c-ANCA and p-ANCA antibodies. Some case reports of GPA and Churg-Strauss syndrome causing retroperitoneal fibrosis have been published in recent years describing the association between the condition and the diseases [13]. It is important to note that the first clinical manifestation of GPA can be retroperitoneal fibrosis. If symptoms develop quickly very few classical GPA signs can be seen.

Some cases have reported similar presentations. Lillaz et al. reported the case of a patient presenting with unilateral renal colic, first considered to be of infectious etiology. Examinations showed a periureteral mass that was causing ureteral compression. A double-J stent was used to resolve the hydronephrosis. The lesion was later surgically removed due to suspicion of a neoplastic process and GPA was diagnosed with histopathology of the removed tissue [14]. Other reported cases were misdiagnosed as tuberculosis and treated with antituberculous drugs until the onset of more classical GPA symptoms revealed the actual disease [15]. With sufficient evidence and a similar treatment regimen for IgG4, GPA, and other vasculitis-induced retroperitoneal fibrosis, preliminary treatment can be considered with cyclophosphamide and corticosteroids and can be adequately altered if needed when the diagnosis is narrowed down further [16].

Inactive fibrosis with a suitable treatment regimen usually remains at least partially present, albeit constant in size after the initial reduction. We have no reason to expect a different outcome with the retroperitoneal fibrosis of our patient. Sometimes, the mass is treated surgically or using stents to prevent the passing structures from collapsing. As our patient currently has no symptoms, has shown improved laboratory findings, and has an improved kidney function, he will continue his treatment at our rheumatology department. Because a satisfactory resolution of the hydronephrosis has been proven, radical treatment has been postponed and the patient will be periodically monitored.

## Conclusions

In our case, cavitating lesions in the lungs combined with confirmed c-ANCA and PR3-ANCA antibodies quickly narrowed down the list of possible diagnoses and pointed clinicians toward GPA. Precise radiological readings and interpretation lowered the suspicion of an abdominal neoplastic process with dissemination into the lungs. Consequently, the time until first treatment and the beginning of disease remission was reduced.

Every clinician should consider GPA as a possible diagnosis for a patient presenting with pulmonary lesions and retroperitoneal fibrosis. In the case of our patient, the fibrosis was centered around the iliac artery and compressed the left ureter. To our knowledge, no such case has yet been described in the literature. Clinicians often reported having trouble recognizing this atypical presentation of GPA before other symptoms presented and delayed the optimal treatment as a result. This case report should contribute to a better understanding of unusual presentations of GPA. We conclude that further research is needed to better understand the causes that lead to retroperitoneal fibrosis.
